# Supplementary material for: XPO1 Expression Is a Poor-Prognosis Marker in Pancreatic Adenocarcinoma
Source: J Clin Med. 2019 Apr 30;8(5):596. doi: 10.3390/jcm8050596 (PMC6572621; doi:10.3390/jcm8050596)
Supplement: Supplementary file 1 [file jcm-08-00596-s001.zip › jcm-486984-supple/20190208_FigS1.pptx]

## Slide 1
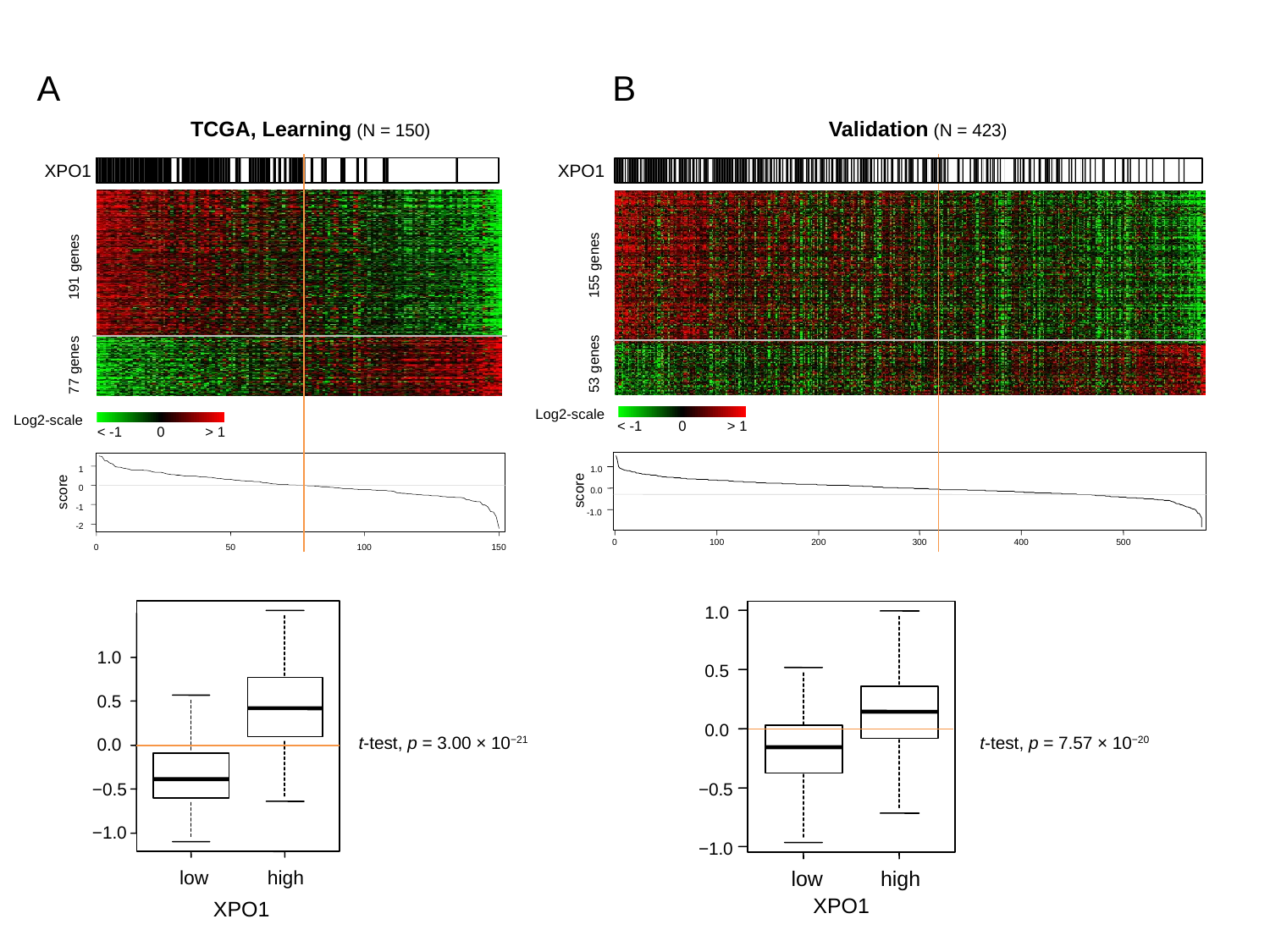

A
B
Validation (N = 423)
TCGA, Learning (N = 150)
XPO1
XPO1
155 genes
191 genes
53 genes
77 genes
Log2-scale
Log2-scale
0
< -1
> 1
0
< -1
> 1
1.0
0.0
score
-1.0
0
100
200
300
400
500
1
0
score
-1
-2
0
50
100
150
1.0
0.5
0.0
−0.5
−1.0
 low
high
XPO1
1.0
0.5
0.0
−0.5
−1.0
low
high
XPO1
t-test, p = 3.00 × 10^−21^
t-test, p = 7.57 × 10^−20^
